# Deep sequencing detects human papillomavirus (HPV) in cervical cancers negative for HPV by PCR

**DOI:** 10.1038/s41416-020-01111-0

**Published:** 2020-10-06

**Authors:** Laila Sara Arroyo Mühr, Camilla Lagheden, Jiayao Lei, Carina Eklund, Sara Nordqvist Kleppe, Pär Sparén, Karin Sundström, Joakim Dillner

**Affiliations:** 1 Center for Cervical Cancer Prevention, Department of Laboratory Medicine, Karolinska Institutet and Karolinska University Laboratory, Karolinska University Hospital, Stockholm, Sweden; 2 Department of Medical Epidemiology & Biostatistics, Karolinska Institutet, Stockholm, Sweden

**Keywords:** Infectious-disease diagnostics, Viral infection, Cervical cancer

## Abstract

**Background:**

Human papillomavirus (HPV) is a necessary cause of cervical cancer, although some invasive cervical cancers may test negative by HPV PCR. We previously requested all invasive cervical cancers in Sweden during 10 years and subjected them to PCR. We also optimised methods for deep sequencing of formalin-fixed paraffin-embedded samples.

**Methods:**

Using Novaseq 6000, we simultaneously sequenced total DNA and cDNA from 392 HPV PCR-negative cervical cancers. Non-human reads were queried against all known HPVs. The complete database now contains PCR and/or deep sequencing data on 2850 invasive cervical cancers.

**Results:**

HPV sequences were detected in 169/392 of HPV PCR-negative cervical cancers. Overall, 30 different HPV types were detected, but only 5 types were present in proportions above 3% of cancers. More than 92% of tumours were HPV-positive in PCR and/or sequencing (95% confidence interval: 91.1–93.1%). Exploring possible reasons for failure to previously detect HPV suggest that more sensitive type-specific PCRs for HPV 31, 33, 45 and 73 targeting retained regions of HPV would have detected most of these (117/392).

**Conclusions:**

Unbiased deep sequencing provides comprehensive data on HPV types in cervical cancers and appears to be an important tool for quality assurance of HPV screening.

## Background

While human papillomavirus (HPV) is a necessary cause for cervical cancer, several authors have reported a proportion of invasive cervical cancers as being HPV negative. The range of HPV negativity varies between studies: in some cervical cancer cohorts, just 0.03% cervical tumours have no HPV detected while others report up to 15% cervical cancers being HPV negative.^1–3^

Possible explanations for not finding HPV among cervical cancers include: cancers independent of HPV (rare true negatives), loss of HPV genomes, cancers caused by HPV types not tested for, misclassification of cancers (e.g. metastasis from other tumours or cancers of corpus uteri misclassified as cervical) and failure of HPV detection methods.^1–4^

To optimise cervical screening, the risk of false negativity in HPV screening needs to be minimised at all stages. HPV-test negative cervical cancers may constitute a biologically distinct subgroup, associated with symptomatic detection, late-stage diagnosis and worse prognosis^5^ that may need different targeted therapeutic strategies.^6^

Common methods to detect HPV are based on PCR and probe hybridisation, usually targeting the *L1* gene, which is the most conserved gene within the HPV genome.^7^ These PCR methods are both efficient and sensitive but are unable to detect HPVs that do not bind specifically to designed primers and probes. An HPV genotype that diverges in its genomic sequence from the sequences designed for primers/probe may escape amplification and/or hybridisation, and therefore remain undetected.^8–10^ HPV detection failure can be readily circumvented by subjecting specimens to unbiased high throughput sequencing of the total nucleic acids of the sample, as HPV can then be detected without prior knowledge/assumptions of which genotype-specific sequences may be present.^8,11,12^ Furthermore, if cDNA is sequenced, the data will show if there is viral transcriptional activity, which is typically essential for both initiation and maintenance of the malignant phenotype.

We thus aimed to assess whether unbiased deep sequencing might detect HPV among cervical cancer specimens testing HPV negative by PCR.

## Methods

### Sample collection

HPV genotyping was previously performed based on *L1* amplification and consequent probe hybridisation on formalin-fixed paraffin-embedded (FFPE) blocks from all cervical cancers occurring during a 10-year period (2002–2011) in Sweden.^13^ In case of HPV negativity, the slide from each case-block was re-reviewed by a pathologist to confirm presence of tissue representative of cervical cancer. FFPE cervical blocks were thereafter subjected to real-time PCR (rt-PCR) for HPV 16 and 18 targeting *E6* and *E7* genes of the HPV genome. Out of the 2850 cervical cases, we reported that almost 14% (394/2850) were HPV negative by PCR.^13^

For this study, we included all HPV negative FFPE blocks (*n* = 394) together with 59 randomly selected HPV positive blocks to be used as positive controls, to assess the overall presence of HPV using an unbiased (i.e. not based on PCR) approach with deep sequencing. As negative controls, we selected all corresponding empty paraffin blocks, “blank blocks”, that had been sectioned in-between each cervical cancer FFPE block (*n* = 453).

A total of 34/59 HPV positive blocks and 92/394 cancers negative by HPV PCR had been already sequenced as part of a methods optimisation paper.^14^ To obtain a comprehensive result of the HPV status in invasive cervical cancers in Sweden during a 10-year period, the previous PCR data and the previous sequencing data were combined with the new sequencing performed here.

### Deep sequencing

All FFPE blocks (*n* = 906) were extracted as previously described.^15^ Cervical FFPE blocks were divided randomly into 7 groups for sequencing. The blank blocks corresponding to the cases in each group, were divided and pooled into 2 negative controls. As a result, each group contained approximately 65 cervical blocks (HPV positive and negative cervical blocks) and 2 negative controls (containing each of these controls, a pool with extracted material from half of the empty paraffin blocks corresponding to the FFPE cases within that group).

For each sample, 8 μl of extracted material was reverse-transcribed, indexed and rRNA-depleted following the SMARTer® Stranded Total RNA-Seq Kit v2 - Pico Input Mammalian library preparation guide (Takara, US), omitting the fragmentation step. The libraries were validated, normalised to 2 nM and pooled according to the 7 groups division before sequencing. RNA seq was performed on NovaSeq 6000 system (Illumina, USA) at 2 × 150 bp, in seven different runs (one run per group), aiming for 30 M paired end reads/sample.

### Bioinformatics

Indices, included in the Illumina adaptors, were used to assign raw sequence reads obtained from the NovaSeq 6000 (Illumina) platform to the originating samples. Reads were quality- and adaptor-trimmed with Trimmomatic^16^ using default parameters and 18 bp as minimal read length. The first 3 nucleotides from every R2 read were trimmed, as advised within the SMARTer pico kit used for library preparation, and thereafter high-quality paired reads were screened against the human reference genome GRCh38 using NextGenMap v.0.5.2.^17^ Reads were filtered out if they aligned with >95 % identity over 75 % of their length to the human genome. Non-human reads were queried against all HPV protein sequences included in the PaVE database (Papillomavirus Episteme, accessed on 28 July 2019, including all protein sequences from HPV reference and non-reference genomes), using the open source software Diamond^18^ blastx with default parameters and –top 1. Samples were considered positive for HPV (cut-off) if a minimum of 10 reads were detected for any HPV type with at least 90 % identity, and a coverage of >10% of that HPV genotype (approx. 790 bp) was present.

### Analysis of the MGP region

Cervical samples classified as positive with deep sequencing which presented HPV genotypes that should have been but were not previously detected by PCR, were subjected to further analysis to elucidate reasons for the false negativity. We analysed the genomic sequence and coverage of HPV reads within the MGP region in the *L1* gene, which is the PCR region targeted for genotyping using a modified general primer (MGP) system,^19^ as well as possible mismatches with primers and probes used in the genotyping method.

Non-human reads were aligned to an HPV database comprising all HPV genomes officially established by the International HPV Reference Center (*n* = 221), https://www.hpvcenter.se, accessed on 2020–01–20), together with complete genome sequences from HPV types that are not officially established yet (*n* = 222, https://pave.niaid.nih.gov, accessed on 2020–01–20), using NextGenMap,^17^ and >90% identity over 75% of read length as selected parameters for alignment. HPV-aligned reads from each sample were thereafter subjected to visual inspection using Integrative Genomics Viewer to confirm mapping of reads within the corresponding genotype, assess coverage and SNPs existence.

### Confirmation of presence of HPV types by rt-PCR

Rt-PCR was used to confirm presence/absence of HPV types in case of discrepancies between PCR-based genotyping and deep sequencing or when a blank block had tested positive. In the cancer cases where this happened, all cervical tumour samples, together with all individual empty paraffin blank blocks within the sequencing run, were subjected to rt-PCR. The rt-PCR was performed at 20 µL reaction volume containing 50–100 ng genomic DNA, 1× TaqMan Universal PCR Master Mix (Applied Biosystems) including 0.5 µM of each forward and reverse primer and 0.25 µM HPV MGB-probe. Water was used as non-template control in each run. The PCR reaction was carried out in ABI 7300 Real-time PCR System, using the Software version 2.0.5 (Applied Biosystems), with the following temperature settings: 10 min at 95 °C, followed by 45 cycles at 95 °C for 15 s and 60 °C for 1 min. The threshold was set to 0.1 ∆Rn (Rn is the fluorescence of the reporter dye divided by the fluorescence of the passive reference. For ∆Rn the baseline fluorescence has been subtracted).

When there was a disagreement in genotyping results between the PCR based method and sequencing, the rt-PCR mixtures contained a total of 20 µL: 5 µL sample and 1× of TaqMan Fast Advanced Master Mix (Applied Biosystems) including 0.5 µM of each primer and 0.25 µM HPV probe. Water was used as non-template control in each run. The PCR analyses were carried out in QuantStudio 3, using the QuantStudio Design & Analysis Software v.1.4.3 (Applied Biosystems), with the following temperature settings: 20 min at 95 °C, followed by 45 cycles at 95 °C for 1 min and 60 °C for 20 s.

## Results

Among all the cases of invasive cervical cancer in Sweden over a 10-year period, we could perform HPV genotyping with PCR and/or deep sequencing for 2850 cases. All FFPE tumour blocks and their corresponding blank paraffin blocks, were first subjected to HPV genotyping. If HPV negative (*n* = 483), the FFPE blocks were then subjected to HPV 16 and 18 rt-PCR targeting *E6* and *E7* genes of the HPV genome. In case still being HPV negative (*n* = 394), the samples were then deep sequenced following an unbiased approach, not based on PCR. A consort diagram can be found in Supplementary Fig. 1.

The median age at cancer diagnosis of the HPV negative cervical cases (*n* = 394) was 62.5 years (range 24–95 years); most women were diagnosed at age 60 or above. Sixty-five percent of the HPV negative cervical cancers were squamous cell carcinoma, 23 % were adenocarcinoma and 12 % were less common histological types, such as adenosquamous cell carcinoma and other rare types. Compared to cases positive for HPV, HPV negative cases were more likely to be diagnosed at older age and more advanced stage (Table 1).

**Table 1:** Characteristics of women with a primary invasive cervical cancer diagnosis 2002–2011 in Sweden by HPV status.

Characteristics	PCR+ *n* = 2456	PCR− *n* = 394		
		PCR− *n* = 394	PCR-/RNAseq− *n* = 223*	PCR-/RNAseq+ *n* = 169*
Age, median (range)	49 (18–98)	62.5 (24–95)	68 (30–93)	57 (24–95)
Age at cancer diagnosis				
<29	165 (6.7)	2 (0.5)	0 (0.0)	2 (1.2)
30–44	860 (35.0)	73 (18.5)	24 (10.8)	49 (29.0)
45–59	608 (24.8)	94 (23.9)	51 (22.9)	42 (24.9)
60–74	428 (17.4)	112 (28.4)	70 (31.4)	42 (24.9)
>74	395 (16.1)	113 (28.7)	78 (35.0)	34 (20.1)
FIGO stage				
IA	473 (19.3)	57 (14.5)	19 (8.5)	38 (22.5)
IB	1027 (41.8)	130 (33.0)	71 (31.8)	59 (34.9)
II	502 (20.4)	75 (19.0)	40 (17.9)	34 (20.1)
III+	454 (18.5)	132 (33.5)	93 (41.7)	38 (22.5)
Histological types				
Adenocarcinoma	437 (17.8)	91 (23.1)	83 (37.2)	7 (4.1)
Adenosquamous cell carcinoma	97 (3.9)	22 (5.6)	21 (9.4)	0 (0.0)
Other types	68 (2.8)	24 (6.1)	22 (9.9)	2 (1.2)
Squamous cell carcinoma	1854 (75.5)	257 (65.2)	97 (43.5)	160 (94.7)

*FIGO* International Federation of Gynecology and Obstetrics.

*There were 2 specimens that had been HPV-negative by PCR but, had too little material available for sequencing and thus only 392/394 HPV-negative cancers were sequenced.

There were two specimens that had been HPV-negative by PCR but, had too little material available for sequencing and thus only 392/394 HPV-negative cancers were sequenced. We also sequenced 59 HPV PCR-positive cervical cancers, selected at random, for comparability of methods.

The NovaSeq sequencing generated high-quality sequencing data, with a median of 2898 million paired reads per run and 30 million paired reads per sample. Most reads (median 96%) were classified as human sequences. We detected HPV sequences in 54/59 HPV PCR-positive cancers, with total concordance regarding the HPV type detected in 46/59 (77.97%) specimens (Table 2), partial concordance in 3/59 specimens (5.08%), and HPV positivity but with a different genotype than detected by PCR in 5/59 (8.47%) specimens (Table 2). Five specimens were HPV negative by sequencing.

**Table 2:** HPV types detected with RNAseq in PCR positive specimens.

	HPVs detected with RNAseq												
	6	16	18	31	33	35	40	45	58	68	73	202	Negative
HPVs detected with PCR													
6													1
16		26*		1						1	1	1	4*
18			12										1
31				1									
33					4								
35					1								
45								4*					1
58									2				
Negative							1*						

*Multiple HPV infection detected. Two PCR positive samples (one case presenting HPV 16 and another case presenting HPV 6/45) revealed presence of HPV 16/40, and only HPV 45 respectively, when subjected to RNAseq.

HPV sequences were detected in 169/392 HPV PCR-negative specimens, with all samples presenting a single infection. HPV 33, 73, 31 and 45 were detected in 117/392 of all HPV PCR negative cervical cancer cases (58/392, 23/392, 19/392 and 17/392 specimens, respectively) (Table 3).

**Table 3:** HPV detection using RNAseq in HPV PCR negative specimens.

	*n* (Total 392)	% Total (95% CI)
HPV Negative by RNAseq	223	56.89 (51.82–61.85)
HPV Positive by RNAseq	169	43.11 (38.15–48.18)
**HPV 16**	4	1.02 (0.28–2.59)
**HPV 18**	1	0.26 (0.01–1.41)
**HPV 31**	19	4.85 (2.94–7.47)
HPV 32	1	0.26 (0.01–1.41)
**HPV 33**	58	14.80 (11.43–18.70)
HPV 34	3	0.77 (0.16–2.22)
**HPV 35**	3	0.77 (0.16–2.22)
HPV 38	1	0.26 (0.01–1.41)
**HPV 39**	8	2.04 (0.89–3.98)
**HPV 42**	2	0.51 (0.06–1.83)
**HPV 45**	17	4.34 (2.55–6.85)
**HPV 52**	2	0.51 (0.06–1.83)
**HPV 53**	2	0.51 (0.06–1.83)
**HPV 54**	1	0.26 (0.01–1.41)
**HPV 56**	8	2.04 (0.89–3.98)
**HPV 58**	8	2.04 (0.89–3.98)
**HPV 59**	4	1.02 (0.28–2.59)
**HPV 66**	1	0.26 (0.01–1.41)
**HPV 68**	1	0.26 (0.01–1.41)
**HPV 70**	1	0.26 (0.01–1.41)
**HPV 73**	23	5.87 (3.76–8.67)
**HPV 82**	1	0.26 (0.01–1.41)

Number of HPV PCR negative specimens that turn HPV positive after being subjected to RNAseq. Highlighted with bold HPV genotypes are the types which present specific probes for the PCR-based detection method.

Combining the deep sequencing results from this paper with previously reported results^13,14^ to provide a comprehensive overview of the presence of specific HPV types in cervical cancers in Sweden found some type of HPV in 92.17% (2625/2848) of all cervical cancers cases (Table 4). There was a total of 30 different HPV types detected but only five types (HPV 16/18/45/33 or 31) were found in >3% of cancers (Table 4).

**Table 4:** HPV status in 2850 cervical cancer cases from a nationwide audit procedure, listed by type (single infections only), multiple, negative and not tested respectively, as defined by PCR, and/or RNA sequencing results.

HPV type	No of cases (*n*)	% of valid results % (95% CI)	% among any HPV infections % (95% CI)
16	1401	49.16 (47.31–51.01)	53.37 (51.44–55.29)
18	460	16.14 (14.81–17.54)	17.52 (16.09–19.03)
45	192	6.74 (5.84–7.72)	7.31 (6.35–8.38)
33	105	3.68 (3.02–4.44)	4.00 (3.28–4.82)
31	88	3.09 (2.48–3.79)	3.35 (2.70–4.11)
52	41	1.44 (1.03–1.95)	1.56 (1.12–2.11)
39	30	1.05 (0.71–1.50)	1.14 (0.77–1.63)
56	29	1.02 (0.68–1.46)	1.11 (0.74–1.58)
73	28	0.98 (0.65–1.42)	1.07 (0.71–1.54)
58	26	0.91 (0.60–1.33)	0.99 (0.65–1.45)
35	22	0.77 (0.48–1.17)	0.84 (0.53–1.27)
70	23	0.81 (0.51–1.21)	0.88 (0.56–1.31)
59	18	0.63 (0.38–1.00)	0.69 (0.41–1.08)
6	11	0.39 (0.19–0.69)	0.42 (0.21–0.75)
53	9	0.32 (0.14–0.60)	0.34 (0.16–0.65)
66	9	0.32 (0.14–0.60)	0.34 (0.16–0.65)
42	8	0.28 (0.12–0.55)	0.31 (0.13–0.60)
68	7	0.25 (0.10–0.51)	0.27 (0.11–0.55)
11	5	0.18 (0.06–0.41)	0.19 (0.06–0.44)
67	5	0.18 (0.06–0.41)	0.19 (0.06–0.44)
51	4	0.14 (0.04–0.36)	0.15 (0.04–0.39)
34	3	0.11 (0.02–0.31)	0.11 (0.02–0.33)
90	2	0.07 (0.01–0.25)	0.08 (0.01–0.28)
69	1	0.04 (0.001–0.20)	0.04 (0.001–0.21)
32	1	0.04 (0.001–0.20)	0.04 (0.001–0.21)
38	1	0.04 (0.001–0.20)	0.04 (0.001–0.21)
54	1	0.04 (0.001–0.20)	0.04 (0.001–0.21)
82	1	0.04 (0.001–0.20)	0.04 (0.001–0.21)
87	1	0.04 (0.001–0.20)	0.04 (0.001–0.21)
202	1	0.04 (0.001–0.20)	0.04 (0.001–0.21)
Multiple HPV infection	92	3.23 (2.61–3.94)	3.51 (2.83–4.28)
Any HPV infection	2625	92.11 (91.05–93.07)	100
HPV negative	223	7.83 (6.87–8.87)	NA
Specimen not tested	2	0.07 (0.01–0.25)	NA
Total valid results	2850	100	NA

### Analysis of the MGP region

One hundred and sixty-four cervical cancers HPV-negative by PCR (164/169, 97.04%) together with 5/59 (8.47%) previously classified as positive with HPV PCR, presented HPV genotypes that should have been previously detected by the PCR-based method but were not. Analysis of the sequencing reads present in the MGP region in the L1 gene (the PCR region targeted for genotyping) revealed that 109/169 cancers had reads covering the MGP region, with 72/109 samples showing identical sequence to the corresponding HPV genotype. Samples presenting with sequences that differed from the PCR-targeted region (37/109) did not show more than 2-point substitutions when comparing the reference genotype sequence comprising the forward primer, probe or reverse primer binding regions. Most of them (23/37) had only a single substitution in one of the three binding regions (Table 5).

**Table 5:** MGP coverage in HPV PCR−/RNASeq+ specimens.

HPV	Total Samples	Samples with NO reads in MGP region	Samples with reads in MGP region	Samples with identical sequence as the reference clone within the MGP region	Samples with subst in F primer	Samples with subst in Probe	Samples with subst in R primer	Comments
16	4	2	2	2	0	0	0	
18	1	1	0					
31	20	7	13	10	1 (1)	0	2 (1)	
33	59	7	52	44		2 (1)	7 (1)	1 sample has both
35	3	1	2	0		2 (1,2)		
39	7	4	3	2	1 (1)			
40	1	0	1	1				
42	2	1	1	0			1 (1)	
45	17	17	0					
52	2	1	1	0			1 (1)	
53	2	0	2	2				
54	1	0	1	1				
56	8	3	5	1	4 (1)	1 (1)		1 sample has both
58	8	3	5	2	3 (1)			
59	5	4	1	1				
66	1	1	0					
68	2	1	1	1				
70	1	1	0					
73	24	6	18	4		12 (1)	13 (1)	11 samples have both
82	1	0	1	1				

### Confirmation of presence of HPV types by rt-PCR

We sequenced 14 pools of blank blocks which comprised all empty paraffin blocks that had been sectioned in-between each cervical cancer FFPE block. Twelve pools showed no traces of HPV, 1 pool revealed presence of HPV 85 (a genotype that had not been detected in any of the case specimens of the study) and 1 pool contained 51 reads of HPV 33. Among the 70 samples sequenced within the same run as the HPV 33 positive blank block, there were 16 HPV 33-positive samples.

We, therefore, subjected all 70 cervical tumours and all 70 blank blocks in this particular run to type-specific real-time PCR targeting HPV 33. Primers and probes for HPV 33 have been previously described.^20^ The Rt-PCR confirmed HPV 33 presence in all reported HPV 33 positive samples (16/16) and also found it in one cervical tumour where it had not been found by sequencing. All the individual empty paraffin blank blocks were negative for HPV 33 by rt-PCR.

In the analyses of the five samples where the PCR and the sequencing had found different genotypes, the rt-PCR found the same HPV genotype as found by the sequencing in all 5 cases.

## Discussion

We find that unbiased (not based on methods requiring prior knowledge of the sequences aimed to be detected) deep sequencing of FFPE specimens of invasive cervical cancers provides improved accuracy and detectability, detecting HPV in 169/392 cancers that had been negative by HPV PCR.

Accurate detection of HPV is essential, as false negativity may translate into failure in the HPV-based cervical screening program. All specimens that were included in this study were confirmed by surgical staging, histopathological re-review, and specimen adequacy assessment (beta-globin detection) to rule out the possibility of HPV negativity being due to misclassification of the tumour and/or specimen inadequacy.^13,21^ While a few of the HPV types detected with deep sequencing (HPV 32, 34 and 38) were not tested for with the PCR detection method used, most of the samples (164/169) revealed HPV types, that should have been detected with the PCR based method. Further analysis of the MGP region (region targeted by PCR primers and probe) revealed that many of the PCR HPV negative cervical cancers (60/169) had no sequences present from the L1 region in the samples, which might explain why a PCR method targeting L1 failed to detect the HPV. Thirty-seven samples showed sequence variability in the sequences targeted by primers or probes, although with not more than 2 substitutions in either one primer and/or probe. For the remaining 72 cancers, we cannot explain why the HPV was not detected before. Our PCR method has a sensitivity of 50 international units of HPV 16 and HPV 18 and 500 genome equivalents for the other oncogenic HPV types,^22^ suggesting that the difference may be attributable to the higher sensitivity of the sequencing.

The majority of the HPV PCR negative samples that showed HPV presence after being sequenced contained high-risk or probable high-risk HPV types (161/169, 95.27%), and only 8/169 (4.73% specimens showed presence of low-risk HPV types 32 (*n* = 1), 34 (*n* = 3), 38 (*n* = 1), 42 (*n* = 2), and 70 (*n* = 1), in line with the fact that low-risk HPV types are only occasionally found in cervical cancer.

We report that there are still 223/2850 (7.82%) cervical cancers where HPV was not detected. A previous meta-analysis including 30,848 invasive cervical cancers worldwide reported an increased overall HPV prevalence from 85.9% in studies published from 1990 to 1999 to 92.9% in studies published from 2006 to 2010.^4^ Similar percentages of HPV-negativity in invasive cervical cancers are reported by other authors (7–11%).^1,4,21^

HPV negativity was diagnosed based on both PCR and next-generation sequencing, and not on immunohistochemistry (cytokeratin 5, pan cytokeratin, protein 63, P16 and P53). In this study, we had pre-specified a cut-off of 10 HPV reads and at least 10% HPV genome coverage for a sample to be considered as HPV positive, to avoid possible false positivity. There were some samples that presented a weak signal of HPV (<10 reads and/or <10% of HPV genome coverage according to our definition). Further studies are needed to elucidate the reproducibility and significance of these.

Factors as duration of tissue storage, type of cancer histology, and stage of cervical cancer are reported to correlate with the detectability of HPV in cervical tumours.^23–26^ The HPV prevalence was indeed very high (96.42%) in stage Ia stage, compared to 84.13% in III+ stage, in line with previous publications suggesting that HPV expression can be lost as the stage of the cervical cancer is progressing.^23^

One retrospective study reported that the duration of tissue storage had a statistically significant impact on HPV detection (*p* < 0.005), with cases from the last 30 years showing around 24% HPV negativity and older cases (stored for >60 years) presenting a significantly lower HPV detection rate, with >50% HPV negativity.^25^ Our study comprised tumour blocks obtained during a 10-year period and therefore, no differences are expected among the HPV detection rate due to the different storage time among them.

It is also known that HPV negativity is more common in several cervical cancer histologies. While squamous cancers and adenocarcinomas in situ are hardly ever HPV negative,^26^ adenosquamous tumours show an HPV prevalence of about 86%^24^ and the HPV prevalence among adenocarcinomas varies between the subtypes.^25^ The present study showed an HPV prevalence of 95.40% for squamous cell carcinomas, 84.28% for adenocarcinomas, 82.35% for adenosquamous carcinomas and 76.09% for other rare invasive cervical cancers, again in line with previous literature.

As reported previously, we chose to perform reverse transcription of mRNAs without DNAse treatment to obtain a maximally sensitive combination of cDNA and some genomic DNA as well.^14^ We detected reads with known HPV viral splice junctions in 81/169 (48%) of the HPV-positive cancer specimens (data not shown), showing that at least a part of these FFPE specimens had some viral mRNA present.^14^

Despite the fact that HPV negative cancers do exist, HPV screening is by far the best option to eliminate cervical cancer. Data from randomised controlled trials are very clear on the very low cervical cancer risk after an HPV-negative screening test.^27^ The studies in our paper are well in line with the findings that HPV screening has higher sensitivity than any other alternative screening method.

In summary, we report that when cervical cancers are tested by both PCR and deep sequencing, HPV sequences exist in >92% cervical cancer specimens. Type-specific PCRs targeting retained regions of the HPV genome of the most common HPV types might be useful for optimal sensitivity in HPV screening. In addition, we suggest that deep sequencing of apparently HPV-negative cervical cancers is useful for quality assurance in HPV screening and detection of HPV infections undetected by HPV PCR methods.

## Supplementary information

Supplemental Figure 1

## Data Availability

All the aligned, non-human sequences are available at the Sequence Read Archive (SRA) within the bio-project ID PRJNA563802.
