# Supplementary figures and images for: Deep sequencing detects human papillomavirus (HPV) in cervical cancers negative for HPV by PCR
Source: Br J Cancer. 2020 Oct 6;123(12):1790–5. doi: 10.1038/s41416-020-01111-0 (PMC7722749; doi:10.1038/s41416-020-01111-0)

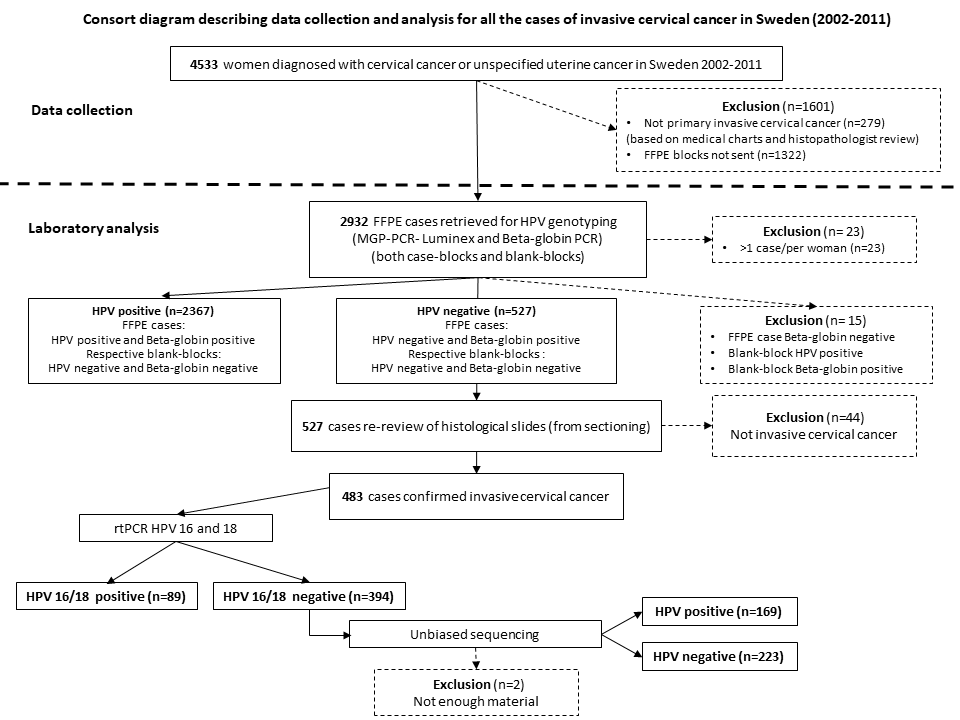

Supplement: Supplementary file 1 — Supplemental Figure 1 [file 41416_2020_1111_MOESM1_ESM.tif]
